# Supplementary material for: Inhibition of DPP4 activity in humans establishes its in vivo role in CXCL10 post‐translational modification: prospective placebo‐controlled clinical studies
Source: EMBO Mol Med. 2016 Apr 14;8(6):679–83. doi: 10.15252/emmm.201506145 (PMC4888857; doi:10.15252/emmm.201506145)
Supplement: Supplementary file 1 — Expanded View Figures PDF [file EMMM-8-679-s001.pdf]

Expanded View Figures

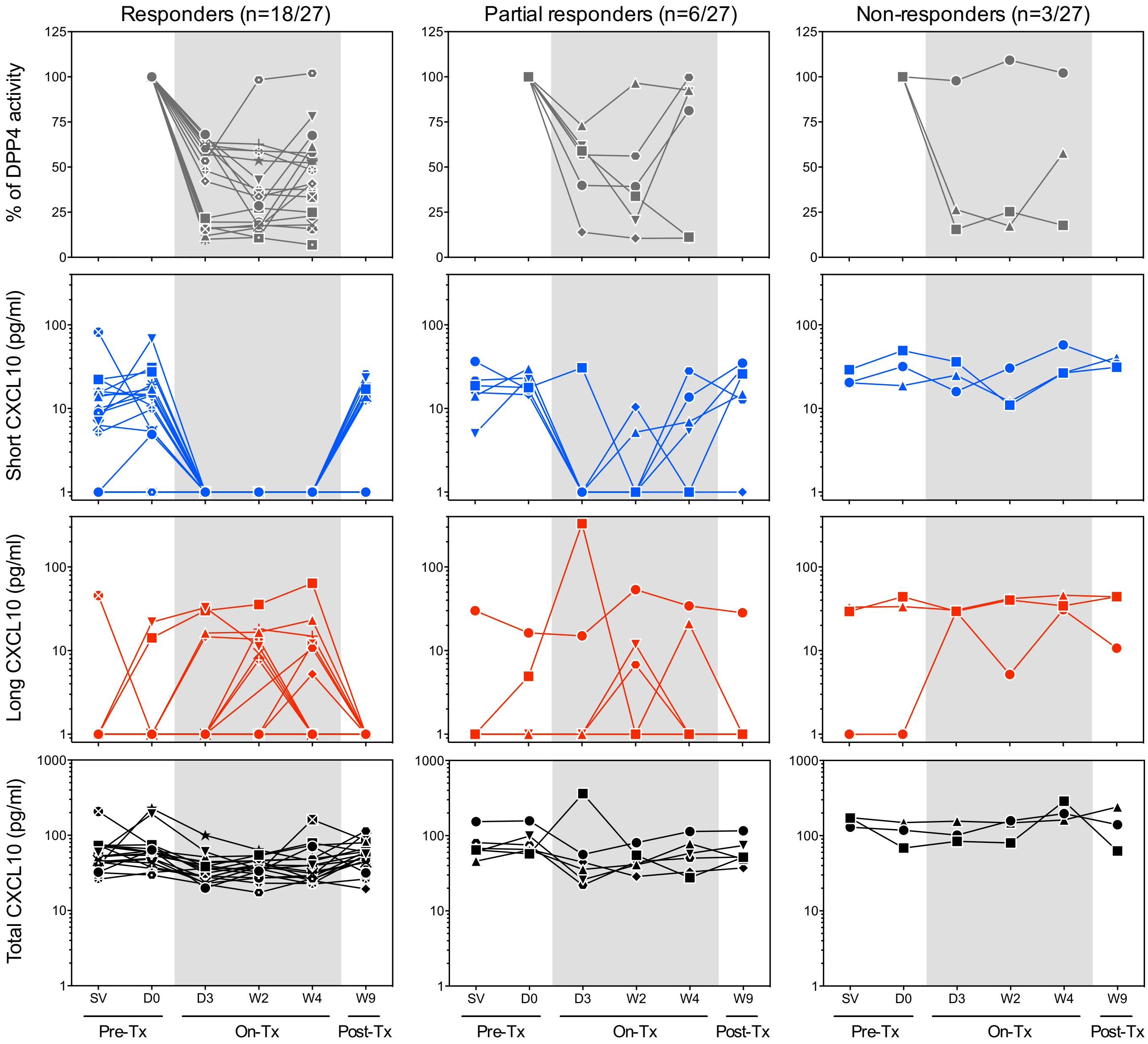

Figure EV1. Detailed follow-up of healthy subjects receiving sitagliptin.
Based on short CXCL10 levels as a pharmacodynamic response to sitagliptin treatment, three categories of subjects were defined: responders ($n = 18$), in which short CXCL10 levels are steadily undetectable upon sitagliptin treatment; partial responders ($n = 6$), which shows a sporadic rise in short CXCL10 upon treatment; and nonresponders ($n = 3$), which shows no change in short CXCL10 levels. For the three groups, the percentage of DPP4 activity and the levels of short, long, and total CXCL10 are shown.

 © 2016 The Authors

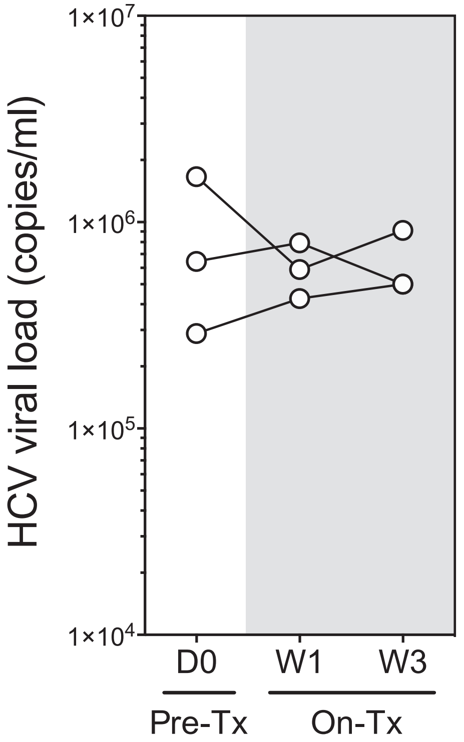

Figure EV2. Chronic HCV patients' viral loads.

© 2016 The Authors
